# Supplementary material for: Replacing protein via enteral nutrition in a stepwise approach in critically ill patients: the REPLENISH randomized clinical trial protocol
Source: Trials. 2023 Jul 30;24:485. doi: 10.1186/s13063-023-07507-6 (PMC10388494; doi:10.1186/s13063-023-07507-6)
Supplement: Supplementary file 2 — Additional file 2: Study proposal. [file 13063_2023_7507_MOESM2_ESM.pdf]

King Abdullah International Medical Research Center
KAIMRC
APPROVED
IRB
Institutional Review Board

King Saud Bin Abdulaziz University for Health Sciences
King Abdullah International Medical Research Center

Replacing Protein via Enteral Nutrition in a Stepwise Approach in Critically Ill Patients: An international, Multicenter Randomized Controlled Trial

(REPLENISH Trial)
Submitted Version: 1, July 08-2019

Version 4.1: June 15, 2022

List of tables and figures

Table 1: Summary of Clinical Practice Guidelines for the protein requirement in critically ill patients

Table 2: Randomized controlled trials examining the association of protein intake and outcomes in critically ill patients.

Table 3: Daily Protein requirement for adults.

Figure 1: Daily protein per kg per day between REPLENISH and standard group.

List of abbreviations

ASPEN	American Society for Parenteral and Enteral Nutrition
AKI	Acute Kidney Injury
APACHE	Acute Physiology and Chronic Health Evaluation
DS MB	Data Safety Monitoring Board
ESPEN	European Society for Clinical Nutrition and Metabolism
EN	Enteral Nutrition
ICU	Intensive Care Unit
KDIGO	Kidney Disease: Improving Global Outcomes
PN	Parenteral Nutrition
RCT	Randomized Controlled Trial
SCCM	Society of Critical Care Medicine
SOFA	Sequential Organ Failure Assessment

Protocol summary

**Title**	**Replacing Protein via Enteral Nutrition in a Stepwise Approach in Critically Ill Patients: An international, Multicenter Randomized Controlled Trial**
**Short Title**	**REPLENISH Trial**
**Design**	An open label, multicenter, international, randomized trial in adult ICU patients.
**Outcomes**	**Primary outcome**: 90 day-all cause mortality
**Intervention**	Adult ICU patients in the two study arms of trial will receive equal amount of energy and protein by the "the primary" enteral formula, in a step-up fashion. Patients will be randomized to receive high protein intake (supplement of 1.2 g/kg/day of enteral protein, added to enteral formula) or standard protein intake (no enteral supplemental protein) from ICU day 5 until ICU discharge up to ICU day 90.
**Sample Size**	2502 mechanically ventilated ICU patients
**Eligibility Criteria**	On ICU calendar day 4 (or on the morning of day 5)<br>1. Age ≥18-years old<br>2. Patient started on EN via nasogastric/orogastric or duodenal or PEG or jejunostomy tubes.<br>3. The patient is on invasive mechanical ventilation and unlikely to be discharged next day.<br>**Exclusion criteria**<br>1. Lack of commitment to full life support or brain death. Patients with "Do Not Resuscitate" order but with commitment to ongoing life support can be enrolled.<br>2. Patients on any amount of parenteral nutrition (PN) in ICU at the time of screening, whether PN is used alone or in combination with enteral nutrition.<br>3. Patients who received an average protein of more than 0.8g/kg/day in the first 4 ICU days.<br>4. Patients being fed entirely through oral route (i.e. those who are eating).<br>5. Pregnancy.<br>6. Burn patients.<br>7. Prisoners or those undergoing forced treatment.<br>8. Patients with hepatic encephalopathy or Child C liver cirrhosis<br>9. Inherited defect of amino acid metabolism.<br>10. Allergies to protein supplement

1. Introduction

The acute phase of critical illness is characterized by a severe proinflammatory response, insulin resistance, gastrointestinal injury and a catabolic state with proteolysis primarily occurring in the skeletal muscles.[1,2] The amino acids released into the circulation from muscles are likely used for tissue repair and synthesis of acute phase proteins and other inflammatory mediators. The resulting protein catabolism may be associated with immunosuppression,[3] poor wound healing[4] and ICU-acquired weakness, which are associated with increased mortality and delayed recovery.[5] Higher protein intake has been thought to mitigate the negative protein catabolic state by increasing the availability of exogenous amino acids. It has been demonstrated that protein breakdown is more pronounced in the early phase of illness which then subsides gradually during the recovery phase.[6] The timing of the switch from the acute phase to the recovery phase of critical illness is probably variable, but may occur at the end the week of critical illness.[6,7]

Table 1: Summary of Clinical Practice Guidelines for the protein supplementation in critically ill patients.

Guideline	Year	Patients	Recommendations	Quality of evidence
National kidney Foundation[8]	2004	Non-ICU patients on maintenance hemodialysis	1.2 g/kg per day	
ESPEN[9]	2006	Conservative therapy<br>Patients with AKI on CCRT<br>Patients with AKI on extracorporeal therapy	0.6–0.8 (max 1.0) g/kg/d<br>up to 1.7 g/kg per day<br>1.0-1.5 g/kg per day	Not rated
Spanish Society of Intensive Care Medicine	2011	General ICU patients	1.2-1.5 g/kg per day	
International Society of Renal Nutrition and Metabolism[10]	2013	Non-ICU patients on maintenance hemodialysis	>1.2 g/kg per day	Not rated
British Dietetic Association[11]	2013	Non-ICU patients on maintenance hemodialysis	A minimum protein intake of 1.1 g/kg ideal body weight per day	
KDIGO[12]	2013	AKI patients not on hemodialysis of CRRT	Suggest to provide protein at 0.8-1.0 g/kg per day and in hypercatabolic patients	Grade 2D
		Patients with AKI on RRT	Suggest to provide protein at 1.0 to 1.5 g/kg per day	Grade 2D
		Patients on CRRT	Suggest to provide protein at up to a maximum of 1.7 g/kg/day	Grade 2D
Canadian Critical Care Society[13]	2015	General ICU patients	There are insufficient data to make a recommendation regarding the use of high protein diets or escalating doses of protein	
ASPEN/SCCM[6]	2016	General ICU patients	1.2–2.0 g/kg/day using actual body weight	Very Low
		ICU patients with AKI not on hemodialysis or CRRT	Standard ICU recommendations for protein (1.2-2 g/kg/day using actual body weight)	Expert consensus
		ICU patients on hemodialysis or CRRT	Increased protein, up to a maximum of 2.5 g/kg/day	Very low
		Liver failure	1.2-2 g/kg/day using dry body weight	Expert consensus
		Burn patients	1.5-2.0 g/kg/day using actual body weight	Expert consensus
		Open abdomen	Additional 15 to 30 grams protein per liter of exudate	Expert consensus
		Obese ICU patients with BMI of 30-40	2.0 g/kg/day using ideal body weight	Expert consensus
		Obese ICU patients with BMI ≥40	2.5 g/kg/day using ideal body weight	Expert consensus
ESPEN[7]	2019	General ICU patients	1.3 g/kg/day using actual body weight can be delivered progressively	Strong consensus
		Obese ICU patients	Protein should be guided by urinary nitrogen losses or lean body mass determination	
			If urinary nitrogen losses or lean body mass determination are not available, protein can be given at 1.3 g/kg/day using adjusted body weight	Consensus

ASPEN/SCCM-American Society for Parenteral and Enteral Nutrition/ Society of Critical Care Medicine; ICU-Intensive Care Unit; CRRT-continuous renal replacement therapy; ESPEN-European Society for Parenteral and Enteral Nutrition

Current clinical practice guidelines have generally recommended higher protein intake in critically ill patients than in healthy individuals (WHO recommendations: 0.7-0.8 g/kg/d),[14] but suggested different protein doses or relatively wide range of protein dose. Protein recommendations from main Clinical Practice Guidelines are summarized in Table 1. The 2016 Society of Critical Care Medicine and American Society for Parenteral and Enteral Nutrition guidelines recommend protein in the range of 1.2–2.0 g/kg actual body weight per day.[15] The 2018 European Society of Clinical Nutrition and Metabolism (ESPEN) recommends 1.3 g/kg protein equivalents per day can be delivered progressively during critical illness.[16]

With the unprecedented pandemic of COVID-19 in early 2020[17], there have been rapid changes in healthcare practices including an increased demand for nutrition support. Initially, 42% patients went on to develop ARDS and remained on ventilator for an average of about 10 days which although more recently decreased but patients experienced hypermetabolism during the acute phase of this infection leading to energy deficit, loss of lean body mass and hypoalbumenemia[18,19]. In addition, these patients are mostly immunocompromised, namely older adults and polymorbid individuals adding to the higher risk of malnutrition risk. A multidisciplinary team of experts including in Lombardy, Italy, the center of the Italian COVID-19 crisis[20] American Society for Parenteral and Enteral Nutrition (ASPEN) Clinical Guidelines Guidelines[21,22] and the European Society for Clinical Nutrition and Metabolism (ESPEN)[23] were put forth for the nutritional care for Pre-ICU and ICU patients with confirmed or suspected COVID-19 infection keeping in view the logistics and safety of the patients as well as the caregiver. They recommend that hypocaloric feeding should be initiated advancing to full dose over the first week of critical illness(70-80% of caloric requirement) along with the protein goal of 1.2-2.0 g/kg ABW/day.

These guidelines are also however largely based on weak evidence and mostly from observational studies that suggested that outcomes are improved with higher protein intake.

1.1 Observational studies

Observational studies showing better outcome with more protein:

Weijs et al. studying 886 patients showed that ICU patients with 1.2-1.5 g/kg/d delivered protein had reduced 28-day mortality.[24] Allingstrup et al showed a stepwise dose-dependent improvement in survival when protein delivery was higher. [25] Nicolo et al found an improvement in survival when patients received >80% of the protein target.[26] Moreover, Compher et al showed that the mortality risk decreased by 6.6% with each 10% increase in protein intake.[27] An observational study demonstrated that the achievement of >90% of target protein intake in early phase of illness was associated with improved ICU outcomes in mechanically ventilated critically ill patients.[28] Weijs et al retrospectively found that early protein intake of ≥1.2 g/kg at day four was associated with better survival in non-overfed non-septic patients on mechanical ventilation (mortality 37% for <0.8 g/kg, 35% for 0.8-1.0 g/kg, 27% for 1.0-1.2 g/kg, and 19% for ≥1.2 g/kg; p=0.033).[29] Another large retrospective cohort study of mixed ICU patients receiving enteral or parenteral nutrition found that early protein intake was associated with increased survival (hazard ratio, 0.83; 95% CI 0.71-0.97, p=0.02) on multivariable Cox regression analysis.[6]

Observational studies showing better outcome with less protein:

There are data suggesting that higher protein intake in early phase of critical illness may actually cause harm, which may be related to inhibition of autophagy and increased ureagenesis.[30,31] Evidence suggests that higher protein delivery in the first week of critical illness might actually be associated with greater muscle wasting[32] and delayed recovery.[33] A pre-planned analysis of the PEPaNIC trial found that higher protein delivery in the first week of critical illness was associated with higher risk of infections and lower risk of earlier live weaning from MV and earlier live ICU discharge.[34] The PROTINVENT (Timing of PROTein INtake and clinical outcomes of adult critically ill patients on prolonged mechanical VENTilation) retrospective study suggested that although an overall low protein intake was associated with the highest mortality risk, high protein intake during the first 3 to 5 ICU days

was associated with increased long-term mortality.[35] Furthermore, amino acid infusion may increase amino acid catabolism in the liver, mediated by increased glucagon, without preventing muscle wasting.[36]

Observational studies showing no difference in outcome with more or less protein:

The Permissive Underfeeding versus Target Enteral Feeding in Adult Critically Ill Patients trial (PermiT) was a randomized controlled trial of restricted caloric intake compared with standard feeding while targeting the full recommended amount of protein in both groups.[37] There was no difference in the primary outcome of mortality in the 2 groups.[37] A subsequent secondary analysis of this trial using propensity score adjustment demonstrated no difference in outcomes among patients who received lower (0.6 ± 0.2 g/kg/day) versus higher protein intake (1.0 ± 0.2 g/kg/day), although the difference in protein between the two groups was moderate.[38]

1.2 Randomized controlled trials

There is scarce evidence from randomized controlled trials that compared higher versus lower protein doses in ICU patients. A meta-analysis of 5 RCTs showed that there was no difference in mortality with the use of higher vs. lower protein.[39] Doig et al randomized 474 adult ICU patients expected to remain in the ICU for >2 days to receive a daily supplement of up to 100 g of IV amino acids or standard care and found no difference in the primary outcome (mean duration of renal dysfunction) and no difference in mortality (or other secondary or tertiary outcomes), although there was a trend towards increased use of renal replacement therapy in patients receiving amino acid therapy (5.5% versus 10.5%, p=0.062).[40] Ferrie et al included 119 patients requiring parenteral nutrition and randomized them to receive 0.8 or 1.2 g/kg parenteral amino acids as part of their nutritional regimen.[41] They found that the patients receiving higher amino acids had less fatigue, greater forearm muscle thickness by ultrasound and better nitrogen balance at day 3, but there was no difference in mortality or length of stay.[41] Rugeles et al. compared hyperproteic (1.4 g/kg/day) hypocaloric with isocaloric (0.76 g/kg/day protein) enteral feeding found significant improvement in SOFA scores at 48 hours in the hyperproteic group.[42] In another study, the same group of investigators administered 1.7 g/kg/day of protein with normocaloric and hypocaloric regimens and did not find any significant differences between the two groups.[43]

Table 2: Randomized controlled trials that examined the association of protein intake and outcomes in critically ill patients.

Study/Year	Population	Inclusion/exclusion criteria	Intervention / comparison	Conclusion / summary
**Clifton -** Enteral hyperalimentation in head injury/1985[44]	20 patients	**Inclusion criteria**<br>Acute severe head injury comatose for at least 24 hours	These patients were randomly placed in two comparable treatment groups: one group was fed with an enteral formula containing 14% of its calories as protein and the other group received a formula containing 22% protein calories	The lower protein group received an average of 26.8 gm/24 hr of nitrogen, equivalent to 188 gm of protein, and the higher protein group 34.3 gm/24 hr, equivalent to 231 gm of protein. Nitrogen balance was -9.2 +/- 6.7 gm/24 hr in the lower protein group and -5.3 +/- 5.0 gm/24 hr in the higher protein group, but the difference did not reach statistical significance
**Doig -** Intravenous amino acid therapy for kidney function in critically ill patients: a randomized controlled trial/2015[45]	474 adult ICU patients	**Inclusion criteria**<br>48 hrs of ICU admission<br>Central line access<br>Able to tolerate 1 lt of fluid<br>>18 yrs<br>**Exclusion criteria**<br>On NSAIDS esp Cox-2 inhibitors<br>On trials involving NO<br>Currently receiving Acetazolamide<br>Patient's current serum creatinine greater than the allowable age and gender<br>Severe Acute Kidney Injury<br>Dialysis / renal replacement<br>Therapy kidney transplant<br>brain dead, Burn, pregnant or currently breastfeeding<br>Previously enrolled<br>Hypersensitivity reaction<br>Inborn error of amino acid metabolism	Critically ill patients were randomized to receive a daily supplement of extra intravenous amino acids up to 100g in comparison to standard care.	There was no difference in the duration renal failure between the two groups. But it did increase the GFR and urine output in the intervention group.
**Geukers -** The effect of short-term high versus normal protein intake on whole-body protein synthesis and balance in children following cardiac surgery: a randomized double-blind controlled clinical trial/2015.[46]	28 children with Congenital heart defect in the pre-operative phase of surgical repair	Inclusion criteria<br>age 3–24 months with CHD<br>**Exclusion**<br>trisomy 21<br>evidence of infection with a fever of more than 35.5^{o} and positive blood culture mechanically ventilated patients.<br>Patients on inotropes not able to tolerate tube feeding<br>Postoprerative use of medication	Patients were randomized to receive protein of 2 g/kg/24 h in the control group and 5 g/kg/24 h in the intervention group. Both the groups received glucose of 6 mg/kg/min<br>Valine kinetics and fractional albumin synthesis rate were measured	There was no difference in the fractional albumin synthesis rate between the two groups. There is increased oxidation and BUN in the High protein group compared to normal protein.
**Ferrie -** Protein Requirements in the Critically Ill: A Randomized Controlled Trial Using Parenteral Nutrition/2016[47]	119 Adult ICU Patients.	All patients requiring PN<br>**Exclusion**<br>< 16 years<br>Not expected to receive 3 days of PN.	Patients were randomized to receive either 0.8g/kg or 1.2g/kg of amino acids. Outcomes measured were Handgrip, Chalder fatigue score and USG for muscle thickness.	Handgrip strength was not different between the two groups except in high amino acid group on the 7th day. They also had less fatigue and more forearm muscles. However, there was no difference in mortality or LOS.
**Allingstrup -** Early goal-directed nutrition versus standard of care in adult intensive care patients: the single-centre, randomized, outcome assessor-blinded EAT-ICU trial/2017[48]	203 patients	**Inclusion criteria**<br>mechanically ventilated ICU patients expected to stay longer than 3 days in the ICU<br>**Exclusion**<br>patients with a BMI equal to or below 17 and those who appeared malnourished	**EGDN group**<br>We estimated nutritional requirements by indirect calorimetry and 24-h urinary urea aiming at covering 100% of requirements from the first full trial day using enteral and parenteral nutrition.<br>**Standard of care group**<br>We aimed at providing 25 kcal/kg/day by enteral nutrition	The EGDN group had less negative energy ($p < 0.001$) and protein ($p < 0.001$) balances in the ICU as compared to the standard of care group. The PCS score at 6 months did not differ between the two groups (mean difference 0.0, 95% CI −5.9 to 5.8, $p = 0.99$); neither did mortality, rates of organ failures, serious adverse reactions or infections in the ICU, length of ICU or hospital stay, or days alive without life support at 90 days.

1.3 Ongoing studies

A number of trials are ongoing which will likely help further understand the optimal protein requirement in different phases of critical illness, in different subgroups and in nutritionally high-risk patients.[49] Below is summary of the major ongoing randomized controlled trials addressing protein intake**;**

NEXIS (Nutrition and Exercise in Critical Illness: A Randomized Trial of Combined Cycle Ergometry and Amino Acids in the ICU) Trial.[50] This trial is funded by National Institute of Health and evaluates the effect of early bedside cycling and intravenous amino acids to a maximum of 2.5 g/kg/day on the physical recovery of the ICU patients. The physical recovery is evaluated by a 6-minute walk test. Other outcomes include muscle mass, muscle strength, functional capacity and quality of life. The sample size is 142 patients.

EFFORT (The Effect of Higher Protein Dosing in Critically Ill Patients) trial.[51] This trial is a large, multicenter, pragmatic, registry-based, randomized clinical trial of 4000 nutritionally high-risk critically ill patients who are randomly allocated to a higher dose of protein (≥2.2 g/kg/day) or usual care (≤1.2 g/kg/day). The primary outcome for this trial is 60-day mortality. Secondary outcomes include time-to-discharge-alive, nutritional adequacy, hospital mortality, readmission to ICU and hospital, and duration of mechanical ventilation, ICU stay, and hospital stay..

TARGET-Protein Trial.[52] This is a prospective, blinded, parallel group, randomized feasibility trial which will enroll patients from ICUs participating in TARGET- The Augmented versus Routine approach to Giving Energy Trial.[53] Patients will receive two liquid nutrient formulae in a blinded fashion with different amounts of protein but similar calories.

1.4 REPLENISH-Pilot study

(Replacing Protein via Enteral Nutrition in a Stepwise Approach in Critically Ill Patients Trial, ClinicalTrials.gov Identifier: NCT03480555).[54] This randomized trial compared high protein (target 1.8 – 2.2 g of protein/kg/day) to low protein (target 0.8 – 1.0 g of protein/kg/day) intake in adult, medical-surgical critically ill patients. Patients received moderate amount of protein and energy till day 5 and then they are randomized on day 5. This feasibility pilot study with sample size of 40 patients was conducted at 3 sites in Saudi Arabia and showed the feasibility of enrolling subjects and achieving separation in protein intake enterally. However, the difference was in the range of 0.6-0.7 g/kg/day (Figure 1). Consequently, we revised the way the protein was administered in this trial to achieve larger separation. In addition, we revised the eligibility criteria, based on recent guidelines and ongoing trials. We also, revised the caloric intake during the intervention to be consistent with current guidelines. We also removed some of the functional outcomes which were found difficult to implement.

Figure 1

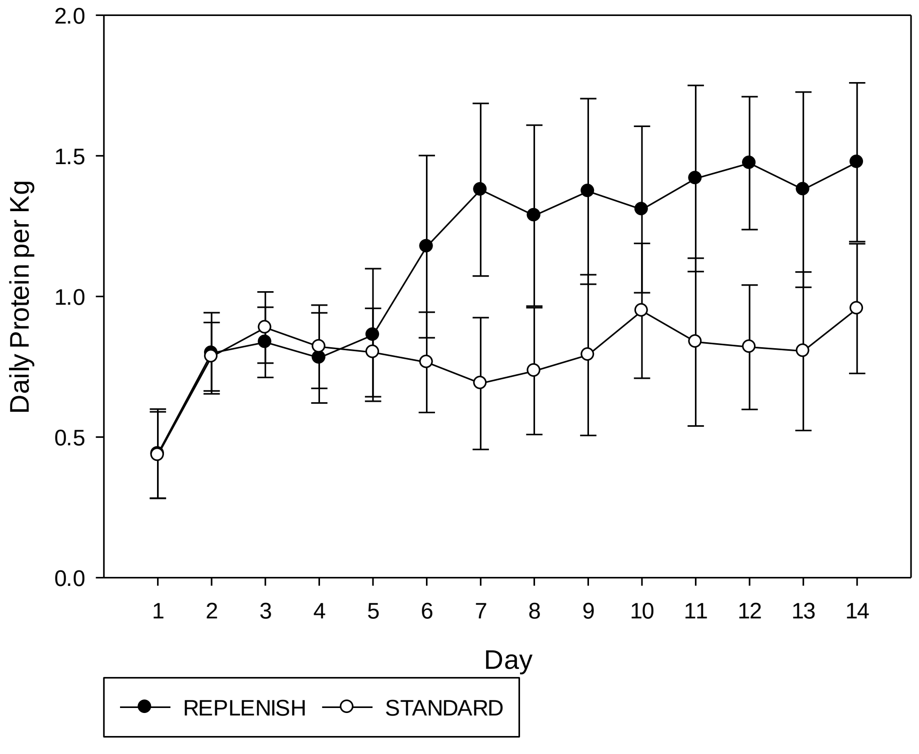

1.5 Rationale for the REPLENISH trial

With the current state of evidence, the optimal amount of protein intake in critically ill patients remains largely unclear and is considered a high priority for research.[2] Interpretation of current studies is complicated by the small sample size, presence of confounders, like increased energy intake in the high protein group in some studies[55] or the heterogenous patient population with varying nutritional risk , who may not respond similarly to the different doses of the protein. Several interventional studies used protein supplementation in the form of intravenous amino acids, not the more widely used enteral protein. Most studies focused on early supplementation of protein. The discrepancy between the results of reported studies calls for further studies on protein intake in ICU patients.[49,56,57] Such studies should employ a more comprehensive approach that integrate the timing and protein target.[16] In addition, there are no dedicated studies on nutrition management in COVID-19 infection and most of the guidelines are based on the best of knowledge and clinical experience. A well-designed randomized multicenter clinical trial to compare higher versus lower amount of protein intake after the acute catabolic phase of critical illness is needed to help resolving the conflicting results of reported studies.[39]

2. Objectives

In adult critically ill patients, does the addition of supplemental enteral protein (1.2 g/kg/day) added to standard formula to achieve high amount of enteral protein (range 2-2.4 g/kg/day) given from ICU day 5 until ICU discharge up to ICU day 90 as compared to no supplemental enteral protein to achieve moderate amount enteral protein (0.8-1.2 g/kg/day), given in conjunction with similar amounts of stepwise caloric administration in the two groups, reduce all-cause 90-day mortality.

3. Methods

3.1 Study design

This is an open label, multicenter, multinational randomized-controlled trial.

Screening:

Subjects will be screened on ICU calendar day 4, up to the morning of ICU calendar day 5. The ICU admission calendar day is considered ICU day 1.

3.2 Study setting

The REPLENISH Trial will be centrally coordinated by Intensive care department, King Abdulaziz Medical city, Riyadh and sponsored by King Abdullah International Medical Research Center. KAIMRC will also be responsible for programming and maintaining the randomization system and database. The study is proposed to be conducted in 20-30 adult general ICUs, both national and international.

3.3 Study population

3.3.1 Inclusion criteria (on ICU calendar day 4 or the morning of day 5)

1. Age ≥18-years old
2. Patient started on EN via nasogastric/orogastric or duodenal or PEG or jejunostomy tubes.
3. The patient is on invasive mechanical ventilation and unlikely to be discharged from ICU next day.

3.3.2 Exclusion criteria

1) Lack of commitment to full life support or brain death. Patients with “Do Not Resuscitate” order but with commitment to ongoing life support can be enrolled.
2) Patients on any amount of parenteral nutrition (PN) in ICU at the time of screening, whether PN is used alone or in combination with enteral nutrition. Non-nutritional calories (dextrose, propofol, citrate) not considered as PN.
3) Patients who received an average protein of more than 0.8g/kg/day in the first 4 ICU days.
4) Patients being fed entirely through oral route – i.e. those who are eating.
5) Pregnancy.

6) Burn patients.

7) Prisoners or those undergoing forced treatment.

8) Patients with hepatic encephalopathy or Child C liver cirrhosis

9) Inherited defect of amino acid metabolism.

10) Allergies to protein supplement

3.4 Recruitment strategy

As per standard practice, the research team will approach the patient if he/she is able to provide informed consent. If not, the surrogate decision maker (SDM) will be approached for consent according to national law. Because both feeding strategies are within the standard of care and because enrollment needs to be done early in order to initiate the feeding strategy, deferred consent can be used if the SDM is not readily available and if legal. If no SDM is present at bedside, the research staff will try to reach him/her by telephone to obtain consent by telephone or will enroll and obtain deferred consent. Consent should be obtained as soon as possible after enteral feeding starting. To facilitate enrollment during weekends, patients can be enrolled anytime in the first 4 calendar days as long as the eligibility criteria are met. The patient will only start the intervention if all inclusion criteria and no exclusion criteria are present on ICU day 4 (or on the morning of day 5).

Once the written informed consent is signed, the patient will be enrolled in the study. In case of deferred consent, written consent should be obtained as soon as possible. Patient can withdraw from the study anytime without any effect on the treatment. The research coordinator will be maintaining a screening log of eligible patients who are not randomized.

3.5 Randomization method

The randomization system will be a 24 x 7 web-based system ensuring allocation of trial participants 1:1 to intervention and control using permuted variable block sizes and stratification by suspected or confirmed COVID-19, trial site and by the use or not of renal replacement therapy at the time of randomization (i.e. the patient at the time of

randomization requires intermittent dialysis or continuous renal replacement therapy, whether or not he/she is actually connected to the machine at that time)

3.6 Coenrollment

Co-enrollment in other RCTs is permitted after approval by both trial steering committees.

3.7 Trial Interventions

3.7.1 Calories

Until ICU calendar day 4, the prescription of energy will be left to the discretion of the treating teams. If desired, energy expenditure can be determined using the predictive equations or indirect calorimetry, based on the practice at individual sites. Between days 5-90, the energy target is 70 to 100% of calculated or measured caloric requirements. Caloric intake will be calculated taking into consideration IV dextrose, citrate and propofol. Caloric intake will include the administered protein in the primary formula.

3.7.2 Protein

Pre-randomization (ICU calender day 1-4)

Until ICU day 4, protein requirement will be provided according to the local practice as long as no intravenous amino acids are given and the average protein intake in the first 4 days does not exceed 0.8 g/kg/day.

Post-randomization (ICU day 5- ICU discharge)

On ICU day 4 (or the morning of day 5), patients will be randomized to;

Standard protein group:

The subjects randomized to this group will receive standard prescription without supplemental proteins (maximum1.2 g/kg/day) from the enteral formula. No supplemental protein will be allowed.

For patients with BMI <30, we will use pre-ICU actual BW for or if unavailable, we will use weight on ICU admission. For patients with BMI >=30, we will use adjusted body weight.[58]

Replenish protein group:

The subjects randomized to this group will receive the standard amount of proteins (maximum 1.2 g/kg/day) from the enteral formula AND supplemental protein at 1.2 g/kg/day. Supplemental proteins will be given as enteral supplements prepared according to manufacturer's recommendation. Supplemental protein may be administered by syringe through the feeding tube with flushs with a minimum of 30-60 ml of water. Supplemental protein may be administered as bolus and the choice of supplemental protein is left to the local teams based on availability .

For patients with BMI <30, we will use pre-ICU actual BW for the calculation; if unavailable, we will use weight on ICU admission. For patients with BMI >=30, we will use adjusted body weight.[58]

Table 3: Daily protein provision for adults.

		ICU Day 1-4	ICU Day 5- ICU discharge or day 90
**Standard protein group**	Calories	Local standards	70-100% of calculated (by equations) or measured (by indirect calorimetry) EE using local standards.
	Protein	Local standards<br>No intravenous amino acids<br>Protein intake on average not to exceed 0.8 g/kg/day	Local standards (maximum1.2 g/kg/day, actual BW for BMI <30 and adjusted BMI for BMI >=30, from the feeding formula.)<br>No supplemental protein
**Replenish protein group**	Calories	Local standards	70-100 % of calculated (by equations) or measured (by indirect calorimetry) EE using local standards.
	Protein	Local standards<br>No intravenous amino acids<br>Protein intake on average not to exceed 0.8 g/kg/day	Local standards (maximum1.2 g/kg/day, actual BW for BMI <30 and adjusted BMI for BMI >=30, from the feeding formula.)<br>**+**<br>Supplemental protein 1.2 g/kg/day actual BW for BMI <30 and adjusted body weight for BMI >=30, given as 4-6 boluses per day or infusion.

Calculation of supplemental protein in the REPLENISH group

1. Calculate BMI
2. If BMI<30, use actual BW. The amount of protein 1.2gX kg of actual BW
3. If BMI >=30, calculate adjusted body weight as follows:
    a. Ideal body weight is computed in men as 50 + (0.91 × [height in centimeters − 152.4]) and in women as 45.5 + (0.91 × [height in centimeters − 152.4])
    b. Calculate adjusted BW= IBW + 0.4 * (Actual weight – IBW)
    c. The amount of protein 1.2g X kg of adjusted BW

3.7.3 Co-interventions

a. **Parenteral nutrition:** Patients on any amount of PN at the time of screening will not be enrolled in the trial. However, patients who have been enrolled in the trial and deemed to need PN by their treating team, will remain in the trial

b. **Glucose management:** All centers are free to use their own standard protocols as long as the target blood glucose is between 4.4-10 mmol/L (80-180 mg/dl).

c. **Mobility assessment:** All centers are free to use their own standard protocols with regards to mobility in ICU patients. However, it will be recorded in the Mobility assessment form

d. Medication data: all data on steroids and statins will be collected during the ICU stay(1-90). If the patient is COVID positive, collect data on prone positioning, ECMO, inhaled nitric oxide, tracheostomy, antivirals and other COVID related medications

3.7.4 Selection of enteral nutritional formulae: The type of enteral formula used will be left to discretion of the attending physician as long as it satisfies the total caloric intake criteria. The types of formula used are grouped as general or disease non-specific (Osmolite, Resourse, Resourse plus, Ensure, Ensure plus, jevity 1.0 and Jevity 1.2) or disease specific (Pulmocare, Glucerna, Suplena, Peptamen 1.0, Peptamen 1.2, Peptamen

1.5, Renal novasource, Nepro, Nutren hepatic, Promote and Vivonex plus). Caloric and protein content of different formulae used in the study are listed in Appendix 1.

3.7.5 The enteral feeding protocol: Each ICU will use their own enteral feeding protocol. The use of prokinetics and type of feeding tube (large-bore nasogastric tube or small-bore nasogastric tube with or without guide wire) is left to the treating team. Patients included in the trial in both arms will receive vitamins as per local practice.

3.8 Randomization, allocation, and intervention arms

3.8.1 Randomization

Patients will be randomized on ICU day 4 up to the morning of day 5 into one of the two intervention arms. The intervention starts on ICU day 5. A list of random blocks will be computer-generated and based on variable block procedure. Randomization will be stratified by suspected or confirmed COVID-19 and then by site and the use of renal replacement therapy at the time of randomization (intermittent hemodialysis or continuous renal replacement therapy).

3.8.2 Allocation

Patient allocation will follow a computer-based concealed process generated by the bioinformatics and research consulting services at King Abdullah International Research Centre (KAIMRC), Riyadh, Saudi Arabia.

3.8.3 Duration of the intervention

The allocated diet and protein supplement will continue until meeting any of the following criteria: death, ICU discharge or day 90 in ICU, premature stopping of feeding due to brain death or palliative care plan whichever comes first, initiation and tolerating of full oral feeding for more than 24 hour (ie. Treating physicians feel that enteral nutrition is no longer required). In these situations, the allocated protocol will no longer be followed but outcome

data will be collected. Upon discharge from the ICU, feeding will be at the discretion of ward clinicians.

3.9 Blinding: This is an open-label study.

3.10 Management of potential risks to participants

a. Adminstration of enteral feeding is a standard practice in ICUs and is not considered to pose any special safety issues. The two levels of protein intake under study (high versus low) are within the recommended ranges of administered protein supplementation to ICU patients by most Clinical Practice Guidelines.
b. Monitoring the kidney and liver functions will be done during the study period as per local standards.

3.11 Outcome measures

I. **Primary outcome:**

   90 day-all-cause mortality

II. **Secondary outcomes:** The secondary outcomes will include:

   a. Days alive at day 90 without life support (use of vasopressor/inotropic support, invasive mechanical ventilation and/or renal replacement therapy)
   b. Days alive and out of hospital at day 90
   c. Bacteremia until 2 days post ICU.
   d. New or progression of Skin Pressure Ulcers on sacral areas in ICU.[59](Appendix 2)
   e. Day 90 Functional assessment using SARC-F screen for sarcopenia.[60] (Appendix 3)
   f. Day 90 functional assessment evaluated by EuroQoL (EQ)-5D-5L) (Appendix 3)

III. **Safety outcomes**

All patients will have a safety evaluation during the intervention period till day 90 in ICU

a. Major safety outcomes will be recorded as one or more of the following 3:

1. New episode of stage 2 or higher AKI by KDIGO criteria after enrollment defined by increase in creatinine 2.0 to 2.9 multiplied by baseline SCr or urine output <0.5 ml/kg/hr for ≥ 12 hours

2. Pneumonia defined as episodes of newly confirmed pneumonia according to the modified CDC criteria

- Two or more serial chest radiographs with at least one of the following (one radiograph is sufficient for patients with no underlying pulmonary or cardiac disease):

  1. new or progressive and persistent infiltrate
  2. consolidation
  3. cavitation

  AND

- At least one of the following:

  1. fever (>38°C) with no other recognized cause
  2. leukopenia (white cell count < 4 x 109 /l) or leukocytosis (white cell count >12 x 109 /l)

  AND

- At least two of the following:

  1. new onset of purulent sputum or change in character of sputum, or increased respiratory secretions or increased suctioning requirements
  2. new onset or worsening cough, or dyspnea, or tachypnea
  3. rales or bronchial breath sounds
  4. worsening gas exchange (hypoxemia, increased oxygen requirement, increased ventilator demand)

3. Grade IV Acute Gastrointestinal injury (AGI) [61]including any of the following:

- Bowel ischaemia with necrosis; defined as any of the following: absent blood flow in one of the main arteries supplying the bowel (superior mesenteric artery, inferior mesenteric artery, or coeliac artery) with evidence of bowel wall compromise on an imaging study (CT angiography, angiography, or magnetic resonance angiography), presence of endoscopy criteria for colonic ischaemia according to the Favier classification system (stage I, petechiae; stage II, petechiae and superficial ulcers; and stage III, necrotic ulcers and polypoid lesions); or evidence of bowel ischaemia during surgery.
- Clinically important gastrointestinal bleeding defined as overt gastrointestinal bleeding and at least one of the following four features within 24 hours of gastrointestinal bleeding (in the absence of other causes) in the intensive care unit a) spontaneous drop of systolic blood pressure, mean arterial pressure or diastolic blood pressure of 20 mmHg or more b) start of vasopressor or a 20% increase in vasopressor dose c) decrease in hemoglobin of at least 2 g/dl (1.24 mmol/l) or d) transfusion of two units of packed red blood cells or more. Overt gastrointestinal bleeding: hematemesis, coffee ground emesis, melena, hematochezia or bloody nasogastric aspirate.
- Ogilvie’s syndrome defined as bowel dilatation if colonic diameter exceeds 6 cm (greater than 9 cm for caecum) or small bowel diameter exceeds 3 cm, diagnosed either on plain abdominal X-ray or CT scan without underlying mechanical obstruction or other organic cause including abdominal distention and pain (80%),

nausea with vomiting (60%), and obstipation (60%) diagnosed with clinical and radiologic evidence [61]

- Abdominal compartment syndrome (ACS) is defined as a persistent intra-abdominal pressure (IAP) of more than 20 mmHg accompanied by new organ dysfunction or failure

b. Minor safety outcomes will be recorded as one or more of the following 3:

1. Feeding intolerance defined as vomiting or large gastric residual volume (GRV) = GRV ≥500 ml/24 h on a single calendar day.
2. Diarrhea is having three or more loose or liquid stools per day with a stool weight greater than 200–250 g/day (or greater than 250 ml/day)[61]
3. Refeeding syndrome is defined as a fall in serum phosphorus below 0.65 mmol/L within 72 hours of the onset of nutritional support and that drop is more than 0.16 mmol/L from a previously recorded reading during the patient's ICU stay[62,63]

4. Data collection

Data will be collected by the assigned research coordinator from day 1 to day 90. Appendix 6 defines the timeline for all the procedures at each follow-up time point.

Time points

4.1 Baseline data (day 1-4):

For ICU day 4 (day of randomization)

a. Demographic information, sex, age,

b. Weight (kg) and height (cms)

c. Admission category, medical, postoperative (non-trauma) and trauma (post-operative and non-operative)

d. Comorbidities,[64](Appendix 5)

e. Pre-morbid Functional assessment using SARC-F screen for sarcopenia.[60]

f. SOFA score day 4

g. Use of neuromuscular blockade infusion, sedative infusion, and steroids.

h. Pre-morbid SARC-F screen for sarcopenia.[60]

i. If the patient suspected or confirmed case of Covid-19, collect procalcitonin, ferritin, IL-6 and lactate

Additionally, for ICU day 1

a. Simplified mortality score (SMS) day 1

b. SOFA score day 1

Nutritional targets (determined before randmization)

Energy target

Protein target from the main formula

Supplemental protein target, if in the REPLENISH Group

4.2 Daily data:

4.2.1 The **following data will be collected** for each ICU day starting day 1 up to day 90 or ICU discharge

1. Calories administered daily

2. Protein administered daily

3. Feeding data regarding the formula type, PN and motility agents used (yes, no)

4. Glucose and insulin data.

5. Vasopressor use (y/n)

6. Use of renal replacement therapy (RRT) (y/n)

7. Use of invasive mechanical ventilation (y/n)

8. Creatinine, BUN and urine output

9. Bed sores(y/n)

10. Bacteremia(y/n)

4.2.2 Safety data collected up to day 90 or ICU discharge (+ 2 day) as outline above

4.3 Other data points on days 1, 4, 7, 14 and 28

4.3.1 Body weight

4.3.2 Mobility assessment: chair, edge of bed, walking

4.3.3

4.3.4 (Optional)

At a subset of participating sites upon their willingness the following data will be collected.

- Lowest p-potassium level.
- Lowest p-magnesium level
- Lowest p-phosphate level
- Body weight
- prealbumin, albumin, nitrogen balance, 24-hour urinary urea, total bilirubin, ammonia, aspartate transaminase (AST), and alanine aminotransferase (ALT). These will be ordered by request for the study patients and will be optional. In case of labs which have multiple readings in a day, worst values will be recorded.

4.4 Frequency and duration of follow-up

Patients will be followed daily until discharge from ICU up to a maximum of 90 days. Hospital discharge date and 90-day outcomes will be documented from the chart or registries or, if need, by telephonic interview from the patient or next of kin if the patient is discharged alive.

90 day follow up will include vital status, date of death if the patient is dead and Functional assessment using SARC-F screen and EuroQoL (EQ)-5D-5L) if the patient is alive at that day.

4.5 Study compliance

Several measures will be taken to ensure optimal compliance with the study protocols. Before launching the study, ICU physicians, nurses and dietitians will attend training sessions, which include mock scenarios about protocols implementation with special emphasis on adjustment of feeding to achieve the caloric intake as per protocol. Follow-up training sessions will be conducted periodically to provide feedback. The Study Steering Committee will meet frequently at the initial stages of the study and as needed thereafter to ensure the correct implementation of the protocols. The adherence to the protocol and data quality will be monitored by the coordinating site on regular basis, at least every three months. Feedback will be provided to each site to further improve adherence to nutritional targets

5. Data management and statistical analysis

Data will be entered via a secure web-based database hosted at KAIMRC, all of which will only be accessible to the study investigators. The database will be password protected and data will be de-identified. Only the data manager, the statistician and the trial SC will have access to all data. Each site will be assigned an identification code. Sites using the paper copy of the data sheets will keep them in a secured cabinet specified specifically for the study. Several procedures to ensure data quality and protocol standardization will also help to minimize bias. These include: 1) a training session will be held for all Research Coordinators from participating centers prior to study commencement to ensure consistency in procedures; 2) A detailed study Instruction Manual will outline each step of the protocol.

5.1 Statistical analysis

5.1.1 Sample size

We anticipate a baseline risk of 90-day mortality 30% and absolute risk reduction of 5% with the protein intervention. The baseline risk was estimated based on a similar cohort from the PermiT trial and SUP-ICU trials. In the PermiT trial which included patients from 7 sites in KSA and Canada, 715 patients received mechanical ventilation for >4 days, and 209 died by day 90 (29.3%). In the SUP-ICU trial, 48% (1571/3282) of all included patients were mechanically ventilated on day 4. Of these, 34% (530/1571) had died on day 90. The SUP-ICU trial enrolled acutely admitted ICU patients with at least one risk factor for GI bleeding in 33 ICUs in 5 countries in Northern Europe. The treatment effect in REPLENISH (5% absolute risk reduction) was based on a propensity-score adjusted analysis which showed an odds ratio for the association of high protein compared to moderate protein of 0.80 (95% CI 0.56, 1.16).

Significance level. Final analysis of the primary outcome will be based on two-sided alpha (α) of 0.05 and power (1- β) = 0.80.

Sample size calculation: Based on these assumptions, we need 1251 patients in each group, 2502 in both groups.

5.1.2 Statistical methods

The analyses will be done in the intention-to-treat population defined as all randomised patients for whom there is consent for the use of data. Baseline characteristics will be summarized as numbers and percentages (categorical variables), whereas the continuous variables will be summarized as medians and IQRs or means and SD.

We will compare the proportions for the primary and secondary outcomes between patients randomized to standard versus Replenish group. We will calculate the relative risk reduction, absolute risk reduction and the number needed to treat to prevent one death. We will present the primary result with a 2-sided p-value (5% level of significance) and 95% confidence intervals (CI) and the secondary results with adjusted CIs. Detailed statistical analysis plan will be developed and published before the last patient is enrolled.

Each component of the composite outcomes, e.g. SAEs and use of life support, will be reported in a supplement to the primary publication, but any differences between these single components will not analysed.

5.2 Interim analysis

In making decision to recommend termination of the study, the Data Safety Monitoring Board shall be guided by a formal stopping rule based on the primary endpoint of 90-day mortality and on the rates of serious adverse events. The interim test statistics shall be primary outcome analysis. It is planned that two equally spaced formal interim analyses will be performed during the monitoring of the study (when 33% and 67% of the sample size has been achieved). The trial may be stopped for safety (based on mortality) ($p<0.01$) or effectiveness (<0.001) or if there is other compelling evidence that trial participants are being harmed. There will be no plans to terminate the trial for futility. We will account for alpha spending by O'Brien Fleming method and the final significance level will be 0.048[65].

5.3 Subgroup analyses

The following a priori subgroups will be analysed:

Medical vs postoperative vs truama patients.

SOFA day 4 stratified a value of 9

Specific diagnoses: sepsis vs not and head injury vs not.

Patients on vasopressors vs those who are not.

KDIGO at enrollment in the 4 groups: 0, 1, 2, 3.

REPLENISH-COVID module (substudy)

In view of the current pandemic, COVID-19 patients will constitute a dominant subgroup. In sites which will allow enrolling these patients who meet the criteria for the study, we will be doing a subgroup analysis that will evaluate the interaction between addition of supplemental enteral protein (1.2 g/kg/day) added to standard formula to achieve high amount of enteral protein (range 2-2.4 g/kg/day) given from ICU day 5 until ICU discharge up to ICU day 90 as compared to no supplemental enteral protein to achieve moderate amount enteral protein (0.8-1.2 g/kg/day), given in conjunction with similar amounts of stepwise caloric administration in the two groups, reduce all-cause 90-day mortality in patients with suspected COVID-19 compared to those who are not.

Following labs will be ordered on day 1, which will be optional for the sites participating in the substudy;

- Lactate (mmol/L)
- Ferritin (ng/mL)
- IL-6 (pg/mL)
- procalcitonin

5.4 Safety analyses

The issue of the safety of critically ill patients is a prime concern in this randomized trial. Several measures are taken to minimize, observe and document any potential safety concerns. First, any unexpected safety concerns will be reported immediately to the Steering Committee. Second, an Independent Data Safety Monitoring Board will be monitoring the safety of the trial (See below). Third, interim analysis will be conducted after recruiting one thirds and two thirds of the total patient number. Since we are not comparing investigational drugs, devices, or procedures and the study is being conducted in ICUs most of the adverse or serious adverse events occur as part of the participants natural disease process. As such, only adverse events that are definitively related to research procedures will be reported as per usual guidelines. As the occurrence of serious adverse events is a trial outcome, these will be continuously reported to the coordinating site and will be part of the 2 formal interim analyses.

5.5 Loss-to-follow up

Patients will be followed post ICU discharge (without further intervention) to document hospital vital status. In occasional randomized patients, the study may be withdrawn by the patient, family, clinical doctor or investigator. In such cases, the patient's data will be included in the group to which they were allocated as per the intention to treat principle and the reason of withdrawal will be documented. If the patient or the family make the withdrawal these will be asked for permission to continue trial data registration. This is done to obtain as much data as possible and produce the most valid trial result.

5.6 Steering committee

The study Steering Committee members will be responsible for overseeing the conduct of the trial, for upholding or modifying study procedures as needed, addressing challenges with protocol implementation, formulating the analysis plan, reviewing and interpreting the data, and preparing the manuscript. This will be achieved through meetings (in-person or by

conference calls) at least quarter yearly. All other committees will report directly to the Steering Committee.

CHAIRMAN

YASEEN ARABI, MD, FCCP, FCCM
Intensive Care Department
King Saud Bin Abdulaziz University for Health Sciences
King Abdulaziz Medical City
Ministry of National Guard, Health Affairs
Riyadh, Kingdom of Saudi Arabia

5.7. Data monitoring committee

Data and safety monitoring boards (DSMB) should provide an independent opinion on the safety and/or efficacy of a product. According to the FDA guidelines, "*An independent DMC is a committee whose members are considered to be independent of those sponsoring, organizing, and conducting the trial*"[66]. Independence is critical for the committee and should be incorporated in all aspects of the operations, functions and reporting of the board. Independence is commonly defined as those who would have no influence in the conduct of the trial or who would not be influenced in any way by the outcome or stopping of a study[67]. The goal of the DSMB correlates to promote ethical research practices, enhance transparency and completeness, hold companies responsible, and to achieve new levels of corporate excellence. The investigators propose contracting an independent DSMB free of financial COIs related to the topic.

6. Study centers and participating sites

The Steering Committee will be responsible for overall management of study, providing central guidance and support to participating centers for protocol adherence. For this trial, we will approach sites from different counties. Presently the following sites have already expressed their interest their particapation.

**International sites**
Belgium<br>Denmark<br>Estonia<br>India
**National sites (KSA)**
King Abdulaziz Medical City, Jeddah<br>King Abdulaziz Hospital, Al Ahsa<br>Prince Mohammed bin Abdulaziz Hospital, Medina<br>King Faisal Specialist Hospital & Research Centre, Riyadh<br>Asir Central Hospital, Asir<br>King Fahad Medical City, Riyadh<br>King Faisal Specialist Hospital & Research Centre, Jeddah<br>Prince Sultan Military Medical City, Riyadh<br>Prince Mohammed bin Abdulaziz, Riyadh<br>King Fahd Hospital of the University-Imam Abdulrahman Bin Faisal University, Dammam

7. Ethical consideration

7.1 Ethics approval

The Coordinating Centre and all participating clinical sites will receive Institutional Review Board (IRB) approval prior to commencing participant enrollment. Depending on local standards, centralized or local IRBs/REBs will approve the study protocol for each site. Before launching the trial, each clinical site will provide the Coordinating Centre with a copy of their ethics approval letter.

7.2 Informed consent

All consecutive eligible patients or their SDM should be approached for participation in the trial. If the patient is unable to provide consent within the time window allowed by the protocol, the research team could also enroll eligible patients and obtain consent subsequently as per local IRB recommendations under a deferred consent model. The objectives of the study and its potential risks and benefits will be explained to the patient or his/her surrogate decision maker by the in-charge research coordinator or a physician who is a part of the research team in a simple and an understandable manner.

7.3 Confidentiality

Information about study participants will be kept confidential and will be managed in accordance with the following rules:

- All study-related information will be stored securely.
- All study participant information will be accessible only to study personnel.
- All paper and electronic CRFs will be identified only by a coded participant number.

If a participant revokes authorization to collect or use personal health information, the clinical site retains the ability to use all information collected prior to the revocation of participant authorization unless otherwise specified.

References

1. Liebau F, Wernerman J, van Loon LJ, Rooyackers O. Effect of initiating enteral protein feeding on whole-body protein turnover in critically ill patients. *Am J Clin Nutr* 2015; **101**(3): 549-57.
2. Arabi YM, Casaer MP, Chapman M, et al. The intensive care medicine research agenda in nutrition and metabolism. *Intensive Care Med* 2017.
3. Fock RA, Blatt SL, Beutler B, et al. Study of lymphocyte subpopulations in bone marrow in a model of protein–energy malnutrition. *Nutrition* 2010; **26**(10): 1021-8.
4. Rai J, Gill S, Kumar BS. The influence of preoperative nutritional status in wound healing after replacement arthroplasty. *Orthopedics* 2002; **25**(4): 417-21.
5. Latronico N, Herridge M, Hopkins RO, et al. The ICM research agenda on intensive care unit-acquired weakness. *Intensive Care Med* 2017.
6. Bendavid I, Zusman O, Kagan I, Theilla M, Cohen J, Singer P. Early Administration of Protein in Critically Ill Patients: A Retrospective Cohort Study. *Nutrients* 2019; **11**(1).
7. Arabi YM, Al-Dorzi HM. Trophic or full nutritional support? *Current opinion in critical care* 2018; **24**(4): 262-8.
8. Beto JA, Bansal VK. Medical nutrition therapy in chronic kidney failure: integrating clinical practice guidelines. *J Am Diet Assoc* 2004; **104**(3): 404-9.
9. Cano N, Fiaccadori E, Tesinsky P, Toigo G, Druml W. ESPEN Guidelines on Enteral Nutrition: Adult Renal Failure$. *Clinical Nutrition* 2006; **25**: 295-310.
10. Ikizler TA, Cano NJ, Franch H, et al. Prevention and treatment of protein energy wasting in chronic kidney disease patients: a consensus statement by the International Society of Renal Nutrition and Metabolism. *Kidney international* 2013; **84**(6): 1096-107.
11. Naylor H, Jackson H, Walker G, et al. B ritish Dietetic Association evidence - based guidelines for the protein requirements of adults undergoing maintenance haemodialysis or peritoneal dialysis. *Journal of Human Nutrition and Dietetics* 2013; **26**(4): 315-28.
12. Kellum JA, Lameire N. Diagnosis, evaluation, and management of acute kidney injury: a KDIGO summary (Part 1). *Critical care* 2013; **17**(1): 204.
13. Critical Care Nutrition. Canadian Clinical practice Guidelines 2015. 2015 http://www.criticalcarenutrition.com/docs/CPGs%202015/Summary%20CPGs%202015%20vs%202013.pdf. Accessed on 28 April.
14. (WHO) WHO. Dietary Reference Intakes for Energy, Carbohydrate, Fibre, Fat, Fatty Acids, Cholesterol, Protein and Amino Acids (Macronutrients) 2007.
15. McClave SA, Taylor BE, Martindale RG, et al. Guidelines for the Provision and Assessment of Nutrition Support Therapy in the Adult Critically Ill Patient: Society of Critical Care Medicine (SCCM) and American Society for Parenteral and Enteral Nutrition (A.S.P.E.N.). *JPEN J Parenter Enteral Nutr* 2016; **40**(2): 159-211.
16. Singer P, Blaser AR, Berger MM, et al. ESPEN guideline on clinical nutrition in the intensive care unit. *Clinical nutrition* 2019; **38**(1): 48-79.
17. World Health Organization. Novel Coronavirus(2019-nCoV). Situation Report - 132 https://www.who.int/docs/default-source/coronaviruse/situation-reports/20200531-covid-19-sitrep-132.pdf?sfvrsn=d9c2eaef_2Accessed 1 June-2020. .
18. Zhou F, Yu T, Du R, et al. Clinical course and risk factors for mortality of adult inpatients with COVID-19 in Wuhan, China: a retrospective cohort study. *Lancet* 2020; **395**(10229): 1054-62.
19. Arentz M, Yim E, Klaff L, et al. Characteristics and Outcomes of 21 Critically Ill Patients With COVID-19 in Washington State. *Jama* 2020; **323**(16): 1612-4.
20. Laviano A, Koverech A, Zanetti M. Nutrition support in the time of SARS-CoV-2 (COVID-19). *Nutrition* 2020; **74**: 110834-.

21. Nutrition therapy in the patient with COVID-19 disease requiring ICU care- updated on April 2020; https://www.nutritioncare.org/uploadedFiles/Documents/Guidelines_and_Clinical_Resources/Nutrition%20Therapy%20COVID-19_SCCM-ASPEN.pdf; Last accessed on 02.06.2020.
22. ASPEN report on nutrition support practice processes with COVID-19: the first response; updated May 2020- https://www.nutritioncare.org/uploadedFiles/Documents/Guidelines_and_Clinical_Resources/COVID19/ASPEN%20Clinical%20Report%20on%20Nutrition%20Support%20Practice%20Processes%20with%20COVID-19%205-26-2020.pdf; last accessed on 02.06.2020.
23. Barazzoni R, Bischoff SC, Breda J, et al. ESPEN expert statements and practical guidance for nutritional management of individuals with SARS-CoV-2 infection. *Clinical Nutrition* 2020; **39**(6): 1631-8.
24. Weijs PJ, Stapel SN, de Groot SD, et al. Optimal protein and energy nutrition decreases mortality in mechanically ventilated, critically ill patients: a prospective observational cohort study. *JPEN J Parenter Enteral Nutr* 2012; **36**(1): 60-8.
25. Allingstrup MJ, Esmailzadeh N, Knudsen AW, et al. Provision of protein and energy in relation to measured requirements in intensive care patients. *Clinical Nutrition* 2012; **31**(4): 462-8.
26. Nicolo M, Heyland DK, Chittams J, Sammarco T, Compher C. Clinical outcomes related to protein delivery in a critically ill population: a multicenter, multinational observation study. *Journal of Parenteral and Enteral Nutrition* 2016; **40**(1): 45-51.
27. Compher C, Chittams J, Sammarco T, Nicolo M, Heyland DK. Greater protein and energy intake may be associated with improved mortality in higher risk critically ill patients: a multicenter, multinational observational study. *Critical care medicine* 2017; **45**(2): 156-63.
28. Song JH, Lee HS, Kim SY, et al. The influence of protein provision in the early phase of intensive care on clinical outcomes for critically ill patients on mechanical ventilation. *Asia Pac J Clin Nutr* 2017; **26**(2): 234-40.
29. Weijs PJ, Looijaard WG, Beishuizen A, Girbes AR, Oudemans-van Straaten HM. Early high protein intake is associated with low mortality and energy overfeeding with high mortality in non-septic mechanically ventilated critically ill patients. *Critical care* 2014; **18**(6): 701.
30. Derde S, Vanhorebeek I, Guiza F, et al. Early parenteral nutrition evokes a phenotype of autophagy deficiency in liver and skeletal muscle of critically ill rabbits. *Endocrinology* 2012; **153**(5): 2267-76.
31. Hermans G, Casaer MP, Clerckx B, et al. Effect of tolerating macronutrient deficit on the development of intensive-care unit acquired weakness: a subanalysis of the EPaNIC trial. *Lancet Respir Med* 2013; **1**(8): 621-9.
32. Puthucheary ZA, Rawal J, McPhail M, et al. Acute skeletal muscle wasting in critical illness. *JAMA* 2013; **310**(15): 1591-600.
33. Casaer MP, Wilmer A, Hermans G, Wouters PJ, Mesotten D, Van den Berghe G. Role of disease and macronutrient dose in the randomized controlled EPaNIC trial: a post hoc analysis. *American journal of respiratory and critical care medicine* 2013; **187**(3): 247-55.
34. Casaer MP, Wilmer A, Hermans G, Wouters PJ, Mesotten D, Van den Berghe G. Role of disease and macronutrient dose in the randomized controlled EPaNIC trial: a post hoc analysis. *Am J Respir Crit Care Med* 2013; **187**(3): 247-55.
35. Koekkoek W, van Setten CHC, Olthof LE, Kars J, van Zanten ARH. Timing of PROTein INtake and clinical outcomes of adult critically ill patients on prolonged mechanical VENTilation: The PROTINVENT retrospective study. *Clin Nutr* 2018.
36. Thiessen SE, Derde S, Derese I, et al. Role of Glucagon in Catabolism and Muscle Wasting of Critical Illness and Modulation by Nutrition. *Am J Respir Crit Care Med* 2017; **196**(9): 1131-43.

37. Arabi YM, Aldawood AS, Haddad SH, et al. Permissive Underfeeding or Standard Enteral Feeding in Critically Ill Adults. *N Engl J Med* 2015; **372**(25): 2398-408.
38. Arabi YM, Al-Dorzi HM, Mehta S, et al. Association of protein intake with the outcomes of critically ill patients: a post hoc analysis of the PermiT trial. *Am J Clin Nutr* 2018; **108**(5): 988-96.
39. Heyland DK, Stapleton R, Compher C. Should We Prescribe More Protein to Critically Ill Patients? *Nutrients* 2018; **10**(4).
40. Doig GS, Simpson F, Bellomo R, et al. Intravenous amino acid therapy for kidney function in critically ill patients: a randomized controlled trial. *Intensive care medicine* 2015; **41**(7): 1197-208.
41. Ferrie S, Allman - Farinelli M, Daley M, Smith K. Protein requirements in the critically ill: a randomized controlled trial using parenteral nutrition. *Journal of Parenteral and Enteral Nutrition* 2016; **40**(6): 795-805.
42. Rugeles S-J, Rueda J-D, Díaz C-E, Rosselli D. Hyperproteic hypocaloric enteral nutrition in the critically ill patient: a randomized controlled clinical trial. *Indian journal of critical care medicine: peer-reviewed, official publication of Indian Society of Critical Care Medicine* 2013; **17**(6): 343.
43. Rugeles S, Villarraga-Angulo LG, Ariza-Gutiérrez A, Chaverra-Kornerup S, Lasalvia P, Rosselli D. High-protein hypocaloric vs normocaloric enteral nutrition in critically ill patients: a randomized clinical trial. *Journal of critical care* 2016; **35**: 110-4.
44. Clifton GL, Robertson CS, Contant CF. Enteral hyperalimentation in head injury. *J Neurosurg* 1985; **62**(2): 186-93.
45. Doig GS, Simpson F, Bellomo R, et al. Intravenous amino acid therapy for kidney function in critically ill patients: a randomized controlled trial. *Intensive Care Med* 2015; **41**(7): 1197-208.
46. Geukers VG, Dijsselhof ME, Jansen NJ, et al. The effect of short-term high versus normal protein intake on whole-body protein synthesis and balance in children following cardiac surgery: a randomized double-blind controlled clinical trial. *Nutr J* 2015; **14**: 72.
47. Ferrie S, Allman-Farinelli M, Daley M, Smith K. Protein Requirements in the Critically Ill: A Randomized Controlled Trial Using Parenteral Nutrition. *JPEN J Parenter Enteral Nutr* 2016; **40**(6): 795-805.
48. Allingstrup MJ, Kondrup J, Wiis J, et al. Early goal-directed nutrition versus standard of care in adult intensive care patients: the single-centre, randomised, outcome assessor-blinded EAT-ICU trial. *Intensive Care Med* 2017; **43**(11): 1637-47.
49. Preiser JC. High protein intake during the early phase of critical illness: yes or no? *Crit Care* 2018; **22**(1): 261.
50. Xu RY, Zhu XF, Yang Y, Ye P. High-sensitive cardiac troponin T. *J Geriatr Cardiol* 2013; **10**(1): 102-9.
51. Heyland DK, Patel J, Bear D, et al. The Effect of Higher Protein Dosing in Critically Ill Patients: A Multicenter Registry-Based Randomized Trial: The EFFORT Trial. *JPEN J Parenter Enteral Nutr* 2019; **43**(3): 326-34.
52. Roldan V, Marin F, Diaz J, et al. High sensitivity cardiac troponin T and interleukin-6 predict adverse cardiovascular events and mortality in anticoagulated patients with atrial fibrillation. *J Thromb Haemost* 2012; **10**(8): 1500-7.
53. Australian TIobot, New Zealand Intensive Care Society Clinical Trials G. Study protocol for the Augmented versus Routine Approach to Giving Energy Trial (TARGET). *Crit Care Resusc* 2018; **20**(1): 6-14.
54. McKie PM, Heublein DM, Scott CG, et al. Defining high-sensitivity cardiac troponin concentrations in the community. *Clin Chem* 2013; **59**(7): 1099-107.
55. Rugeles SJ, Rueda JD, Diaz CE, Rosselli D. Hyperproteic hypocaloric enteral nutrition in the critically ill patient: A randomized controlled clinical trial. *Indian J Crit Care Med* 2013; **17**(6): 343-9.

56. Preiser JC, van Zanten AR, Berger MM, et al. Metabolic and nutritional support of critically ill patients: consensus and controversies. *Crit Care* 2015; **19**: 35.
57. Singer P, Hiesmayr M, Biolo G, et al. Pragmatic approach to nutrition in the ICU: expert opinion regarding which calorie protein target. *Clin Nutr* 2014; **33**(2): 246-51.
58. Singer P, Blaser AR, Berger MM, et al. ESPEN guideline on clinical nutrition in the intensive care unit. *Clin Nutr* 2019; **38**(1): 48-79.
59. http://www.npuap.org/resources/educational-and-clinical-resources/npuap-pressure-ulcer-stagescategories/.
60. Malmstrom TK, Morley JE. SARC-F: a simple questionnaire to rapidly diagnose sarcopenia. *J Am Med Dir Assoc* 2013; **14**(8): 531-2.
61. Reintam Blaser A, Malbrain ML, Starkopf J, et al. Gastrointestinal function in intensive care patients: terminology, definitions and management. Recommendations of the ESICM Working Group on Abdominal Problems. *Intensive Care Med* 2012; **38**(3): 384-94.
62. Doig GS, Simpson F, Heighes PT, et al. Restricted versus continued standard caloric intake during the management of refeeding syndrome in critically ill adults: a randomised, parallel-group, multicentre, single-blind controlled trial. *Lancet Respir Med* 2015; **3**(12): 943-52.
63. Marik PE, Bedigian MK. Refeeding hypophosphatemia in critically ill patients in an intensive care unit. A prospective study. *Arch Surg* 1996; **131**(10): 1043-7.
64. KNAUS WA, DRAPER EA, WAGNER DP, ZIMMERMAN JE. APACHE II: A severity of disease classification system. *Critical Care Medicine* 1985; **13**(10): 818-29.
65. O'Brien PC, Fleming TR. A multiple testing procedure for clinical trials. *Biometrics* 1979; **35**(3): 549-56.
66. Bethseda M. Guidance for clinical Trial Sponsors:on the establishment and operation of clinical trial data monitoring committees. *United States Food and drug Administration* November 2001.
67. Elenberg. Independent data monitoring committees:rationale, operations and controversies. *Statistics in Medicine* 2001; **20**: 2573-83.

Appendix 1

Caloric and protein content of different formulae used in the study (other formulae not listed are also acceptable, and should be documented).

Formula	Manufacturer	Calories Kcal/ml	Composition			Caloric distribution		
			Protein g/100 ml	Carbohydrate g/100 ml	Fat g/100 ml	Protein %	CHO %	Fat %
**Disease-non-specific**								
Osmolite	Abbott	1.06	4.4	14.3	3.5	17	54	29
Jevity	Abbott	1.06	4.4	15.4	3.5	17	54	29
Promote	Abbott	1	6.2	13.0	2.6	25	52	23
Ensure Plus	Abbott	1.5	6.2	20	5	15	57	28
Resource	Nestle	1.05	3.7	14.7	3.7	14	56	30
Ensure	Abbott	1	4	13.6	3.4	16	54	30
Resource plus	Nestle	1.5	5.5	22.6	4.5	15	60	25
Jevity (1.2)	Abbott	1.2	5.6	17.0	3.9	18.5	52.5	29
**Disease-specific**								
Glucerna	Abbott	0.99	4.2	8.1	5.4	17	34	49
Nutren Hepatic	Nestle	1.5	4	29	2.1	11	77	12
Nepro	Abbott	2	7	22	9.6	14	41	43
Pulmocare	Abbott	1.5	6.2	10.5	9.3	17	28	55
Novasource Renal	Nestle	2.0	9.1	18.4	10	18	37	45
Peptamen (1.5)	Nestle	1.5	6.8	18.4	5.6	18	49	33
Peptamen (1.0)	Nestle	1	4.0	12.8	3.9	16	51	33
Suplena	Abbott	2	3	25.6	9.6	6	51	43
Oxepa	Abbott	1.5	7.0	10.5	9.4	17	28	55

Beneprotein, Nestle: one packet (one 7 g serving) contains: 25 Kcal, 6 grams of proteins, 0 grams of CHO and 0 grams of fat.

Appendix 2

The National Pressure Ulcer Advisory Panel (NPUAP) classification

**SKIN ASSESSMENT STAGING (If Normal, please write "0")**	
**Category/Stage I: Non-blanchable erythema**<br>Intact skin with non-blanchable redness of a localized area usually over a bony prominence. Darkly pigmented skin may not have visible blanching; its color may differ from the surrounding area. The area may be painful, firm, soft, warmer or cooler as compared to adjacent tissue. Category I may be difficult to detect in individuals with dark skin tones. May indicate "at risk" persons.	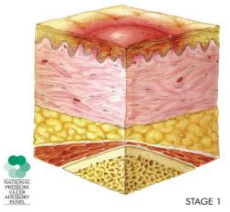
**Category/Stage II: Partial thickness.**<br>Partial thickness loss of dermis presenting as a shallow open ulcer with a red pink wound bed, without slough. May also present as an intact or open/ruptured serum-filled or sero-sanginous filled blister .Presents as a shiny or dry shallow ulcer without slough or bruising*. This category should not be used to describe skin tears, tape burns, incontinence associated dermatitis, maceration or excoriation. * Bruising indicates deep tissue injury.	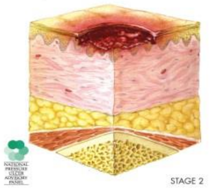
**Category/Stage III: Full thickness skin loss.**<br>Full thickness tissue loss. Subcutaneous fat may be visible but bone, tendon or muscle are *not* exposed. Slough may be present but does not obscure the depth of tissue loss. *May* include undermining and tunneling. The depth of a Category/Stage III pressure ulcer varies by anatomical location. The bridge of the nose, ear, occiput and malleolus do not have (adipose) subcutaneous tissue and Category/Stage III ulcers can be shallow. In contrast, areas of significant adiposity can develop extremely deep Category/Stage III pressure ulcers. Bone/tendon is not visible or directly palpable.	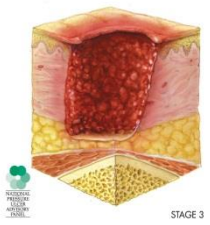
**Category/Stage IV: Full thickness tissue loss.** Full thickness tissue loss with exposed bone, tendon or muscle. Slough or eschar may be present. Often includes undermining and tunneling. The depth of a Category/Stage IV pressure ulcer varies by anatomical location. The bridge of the nose, ear, occiput and malleolus do not have (adipose) subcutaneous tissue and these ulcers can be shallow. Category/Stage IV ulcers can extend into muscle and/or supporting structures (e.g., fascia, tendon or joint capsule) making osteomyelitis or osteitis likely to occur. Exposed bone/muscle is visible or directly palpable.	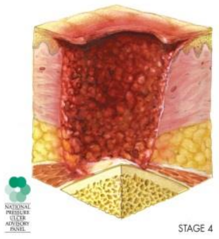
**Unstageable/Unclassified:** Full thickness skin or tissue loss – depth unknown. Full thickness tissue loss in which actual depth of the ulcer is completely obscured by slough (yellow, tan, gray, green or brown) and/or eschar (tan, brown or black) in the wound bed. Until enough slough and/or eschar are removed to expose the base of the wound, the true depth cannot be determined; but it will be either a Category/Stage III or IV. Stable (dry, adherent, intact without erythema or fluctuance) eschar on the heels serves as "the body's natural (biological) cover" and should not be removed.	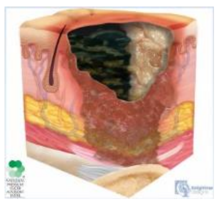
**Suspected Deep Tissue Injury:** depth unknown. Purple or maroon localized area of discolored intact skin or blood-filled blister due to damage of underlying soft tissue from pressure and/or shear. The area may be preceded by tissue that is painful, firm, mushy, boggy, warmer or cooler as compared to adjacent tissue. Deep tissue injury may be difficult to detect in individuals with dark skin tones. Evolution may include a thin blister over a dark wound bed. The wound may further evolve and become covered by thin eschar. Evolution may be rapid exposing additional layers of tissue even with optimal treatment.	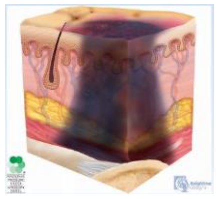

Functional Assessment

Appendix 3

SARC-F screen for sarcopenia

Component	Question	Scoring
Strength	How much difficulty do you have in lifting and carrying 10 pounds?	None = 0<br>Some = 1<br>A lot or unable = 2
Assistance in walking	How much difficulty do you have walking across a room?	None = 0<br>Some = 1<br>A lot, use aids, or unable = 2
Rise from a chair	How much difficulty do you have transferring from a chair or bed?	None = 0<br>Some = 1<br>A lot or unable without help = 2
Climb stairs	How much difficulty do you have climbing a flight of 10 stairs?	None = 0<br>Some = 1<br>A lot or unable = 2
Falls	How many times have you fallen in the past year?	None = 0<br>1–3 falls = 1<br>4 or more falls = 2

Appendix 4
EuroQoL (EQ)-5D-5L

Pre-morbid functional assessment evaluated by EuroQoL (EQ)-5D-5L)

Under each heading, please tick the one box that best describes your health today

MOBILITY

I have no problems in walking about ☐

I have slight problems in walking about ☐

I have moderate problems in walking about ☐

I have severe problems in walking about ☐

I am unable to walk about ☐

SELF-CARE

I have no problems washing or dressing myself ☐

I have slight problems washing or dressing myself ☐

I have moderate problems washing or dressing myself ☐

I have severe problems washing or dressing myself ☐

I am unable to wash or dress myself ☐

USUAL ACTIVITIES (*e.g work, study, housework, family or leisure activities***)**

I have no problems doing my usual activities ☐

I have slight problems doing my usual activities ☐

I have moderate problems doing my usual activities ☐

I have severe problems doing my usual activities ☐

I am unable to my usual activities ☐

PAIN / DISCOMFORT

I have no pain or discomfort .... ☐

I have slight pain or discomfort .... ☐

I have moderate pain or discomfort .... ☐

I have severe pain or discomfort .... ☐

I have extreme pain or discomfort.. ☐

ANXIETY / DEPRESSION

I am not anxious or depressed.. ☐

I am slightly anxious or depressed.. ☐

I am moderately anxious or depressed .... ☐

I am severely anxious or depressed .... ☐

I am extremely anxious or depressed .... ☐

- We would like to know how good or bad your health is TODAY
- The scale is numbered from 0 to 100
- 100 means the best health you can imagine and 0 means the worst health you can imagine
- Mark an X on the scale to indicate how your health is TODAY
- Now, please write the number you marked on the scale in the box below

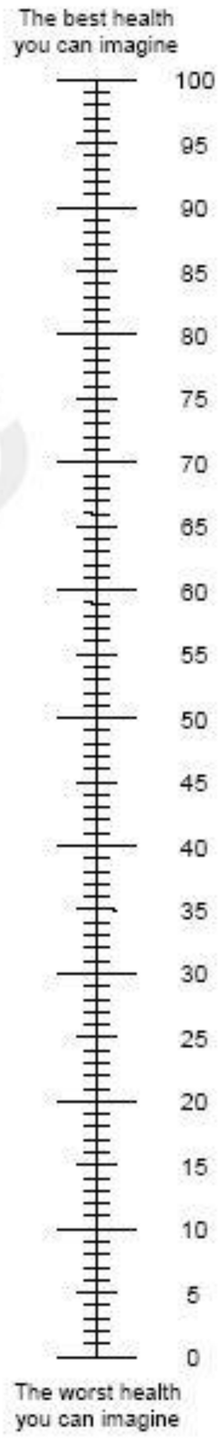

Appendix 5

Comorbidities

APACHE II Definition of Chronic Health Conditions.

Organ insufficiency or immunocompromised state must have been evident prior to this hospital admission and conform to the following criteria:

Comorbidities	Definition
**Liver**	biopsy proven cirrhosis and documented portal hypertension; episodes of past upper GI bleeding attributed to portal hypertension; or prior episodes of hepatic failure/encephalopathy/coma.
**Cardiovascular**	New York Heart Association Class IV (Severe) Unable to carry out any physical activity without discomfort. Symptoms of cardiac insufficiency at rest. If any physical activity is undertaken discomfort is increased.
**Respiratory**	Chronic restrictive, obstructive, or vascular disease resulting in severe exercise restriction (i.e., unable to climb stairs or perform household duties; or documented chronic hypoxia, hypercapnia, secondary polycythemia, severe pulmonary hypertension (>40 mmHg), or respirator dependency.
**Renal**	receiving chronic dialysis.
**Immunocompromised**	the patient has received therapy that suppresses resistance to infection (e.g., immunosuppression, chemotherapy, radiation, long term or recent high dose steroids, or has a disease that is sufficiently advanced to suppress resistance to infection, e.g., leukemia, lymphoma, AIDS).

Appendix 6

	Data At Baseline (Day 1)	Data at Day 4[1]	Daily data from day 1 to day 90, only while in ICU[2]	Weekly data on ICU Days 1, 4, 7, 14, 28 only in ICU[3]	Data from day 1 to day 90 whether in ICU or not [2]	Day 90
Demographics						
Admission category						
Height						
Weight				x		
SMS	x	x				
Comorbidities		x				
INR		x				
Morning blood glucose		x				
SARC-F		x				x
Neuromuscular blockade infusion		x				
sedative infusion		x				
steroids		x				
SOFA		x				
**For COVID patients**						
IL6		x				
Ferritin		x				
Procalcitonin		x				
Lactic acid		x				
Invasive mechanical ventilation: yes, no					x	
Vasopressor: yes, no					x	
RRT: yes, no					x	
Calories: Total: enteral, dextrose, Propofol and citrate			x			
Protein: total: enteral formula, supplemental, parenteral. Supplemental given as bolus vs infusion			x			
Morning blood glucose			x			
Total insulin dose (regular and long acting)			x			
Type of feeding formula			x			
Motility agents (metoclopramide, erythromycin, etc.)			x			
Creatinine			x			
BUN			x			
24-hour urine output			x			
Refeeding syndrome			x			
Diarrhea			x			
Bacteremia			x			
Feeding intolerance			x			
CT scan documented bowel ischemia			x			
GI bleed			x			
Ogilvie's syndrome			x			
Abdominal compartment syndrome			x			
Mortality and date						x
EuroQoL (EQ)-5D-5L						x
Bed sore skin assessment			x			
Mobility assessment				x		
**Optional**						
Lowest blood glucose				x		
Lowest potassium level				x		
Lowest magnesium level				x		
Lowest phosphate level				x		
Prealbumin				x		
Albumin				x		
Blood urea nitrogen (BUN)				x		
Total bilirubin				x		
Transferrin				x		
Nitrogen balance				x		
24-hour urinary urea				x		
Ammonia				x		
AST				x		
Cointervention						
Steroids						
Statins						
For COVID patients						
ECMO						
Inhaled nitric oxide						

Prone positioning						
Tracheostomy						
COVID related medication						

King Abdullah International Medical Research Center
KAIMRC
APPROVED
IRB
Institutional Review Board
